# Determining Connections between the Daily Lives of Zoo Elephants and Their Welfare: An Epidemiological Approach

**DOI:** 10.1371/journal.pone.0158124

**Published:** 2016-07-14

**Authors:** Cheryl L. Meehan, Joy A. Mench, Kathy Carlstead, Jennifer N. Hogan

**Affiliations:** 1 AWARE Institute, Portland, Oregon, United States of America; 2 Center for Animal Welfare, University of California Davis, Davis, California, United States of America; 3 Department of Animal Science, University of California Davis, Davis, California, United States of America; 4 Honolulu Zoo Society, Honolulu, Hawaii, United States of America; University of Florida, UNITED STATES

## Abstract

Concerns about animal welfare increasingly shape people’s views about the acceptability of keeping animals for food production, biomedical research, and in zoos. The field of animal welfare science has developed over the past 50 years as a method of investigating these concerns via research that assesses how living in human-controlled environments influences the behavior, health and affective states of animals. Initially, animal welfare research focused on animals in agricultural settings, but the field has expanded to zoos because good animal welfare is essential to zoos’ mission of promoting connections between animals and visitors and raising awareness of conservation issues. A particular challenge for zoos is ensuring good animal welfare for long-lived, highly social species like elephants. Our main goal in conducting an epidemiological study of African (*Loxodonta africana*) and Asian (*Elephas maximus*) elephant welfare in 68 accredited North American zoos was to understand the prevalence of welfare indicators in the population and determine the aspects of an elephant’s zoo environment, social life and management that are most important to prevent and reduce a variety of welfare problems. In this overview, we provide a summary of the findings of the nine papers in the collection titled: *Epidemiological Investigations of North American Zoo Elephant Welfare* with a focus on the life history, social, housing, and management factors found to be associated with particular aspects of elephant welfare, including the performance of abnormal behavior, foot and joint problems, recumbence, walking rates, and reproductive health issues. Social and management factors were found to be important for multiple indicators of welfare, while exhibit space was found to be less influential than expected. This body of work results from the largest prospective zoo-based animal welfare study conducted to date and sets in motion the process of using science-based welfare benchmarks to optimize care of zoo elephants.

## Introduction

Scientifically addressing questions regarding zoo elephant welfare is timely and relevant because of the broad public interest in the care and management of animals in zoos and aquariums that exists today. This is particularly true for high-profile species such as elephants, which pose management challenges because they are intelligent, highly social, and inhabit large home ranges in the wild. In order to meet these challenges, zoological professionals need objective information about how elephants are faring behaviorally, physiologically and emotionally and how housing and management practices in zoos influence these outcomes both positively and negatively. This collection documents a large-scale collaborative research effort to address these questions with the goals of providing information that can inform subsequent discourse and support evidence-based elephant management in zoos.

We begin this paper with an introduction to the field of animal welfare science, including its origins and methodologies, and then provide context for how the field has evolved within zoological settings. Finally, we provide an integrative synopsis of the results from the nine papers in the collection, highlighting themes and, in some cases, surprises that were revealed by this research.

## Animal Welfare Science- Background and Approaches

Concerns about animal welfare increasingly shape people’s views about the acceptability of keeping animals for food production, biomedical research, and in zoos. Although these kinds of concerns are not new, it was not until the 1960s that significant public interest in ensuring humane treatment of animals became apparent. In the United States concerns centered on the use of animals (mainly dogs and cats) for biomedical research, and led to the passage of the Animal Welfare Act in 1964 [1].

In contrast, in Europe the primary focus was on agricultural animals. In the same year the Animal Welfare Act was passed, Ruth Harrison published a serialized book, *Animal Machine*s [2], in a London newspaper that opened the UK public’s eyes to the realities of modern farm animal production. She coined the term “factory farming” to describe intensive production conditions that involved large numbers of animals housed indoors in enclosures in which their movement and behavior were extremely restricted. Her book created a public outcry and led to the establishment of an influential government investigative committee. That committee’s report broke new ground by stating that both the physical and mental states of animals were important for their welfare, and also recommended that all farm animals be provided at least sufficient space to “stand up, lie down, turn around, groom themselves and stretch their limbs,” principles which became referred to as the “Five Freedoms” [3].

Over time, this list of freedoms was expanded to include freedom from fear, distress, discomfort, pain, injury, disease, hunger, and thirst as well as freedom to express normal behaviors via provision of appropriate facilities and social companions [4]. These “new” Five Freedoms have been accepted by legislative and standards-setting bodies in many countries as ethical principles that underlie the appropriate care and treatment of animals. Importantly for the purpose of this paper and for the development of the research described in this collection, the Five Freedoms and the impetus to use them to evaluate and (where necessary) modify the housing and management of farm animals acted as the stimulus for the development of the field of animal welfare science [5].

Animal welfare science is of necessity multidisciplinary. For the historical reasons mentioned above related to concerns about the restriction of movement and behavior of farm animals, applied animal behavior scientists (ethologists) initially played the major role in the development of the field [6]. While ethology is still a key discipline, many other disciplines now contribute to the increasing body of animal welfare research and research application [5–8] including physiology, veterinary medicine, agricultural and biological engineering, comparative psychology, nutrition, genetics, microbiology, social sciences, and applied ethics.

Broadly, the scientific assessment of animal welfare focuses on animals’ behavior and biological functioning. Because there has been an emphasis on identifying and reducing states of animal suffering in the absence of methods for direct assessment of animals’ feelings, most animal welfare research has been concerned with identifying and validating physiological and behavioral indicators thought to be associated with negative affective states such as frustration, pain, boredom, and fear [5] as well as on understanding the causes of and methods for reducing or preventing the performance of abnormal behaviors like stereotypic behavior [9]. Assessment of biological functioning is generally approached through measurement of physiological responses indicative of stress, such as glucocorticoids [10, 11] as well as the incidence and severity of injury and disease. There is also increasing interest in developing valid measures of positive affect (reviewed [12, 13]) to improve the quality of life for animals by providing them with rewarding experiences. This area of inquiry is in its early stages, but is a promising avenue for future advances in the field.

The measures and methods utilized in studies of animal welfare need to be well matched to the questions of interest and the context of the investigation. For example, some types of animal welfare questions are best studied in controlled settings where specific treatments can be explicitly linked with variation in outcomes, and the vast majority of foundational animal welfare research has been conducted in such settings. However, there is increasing interest in applying large-scale non-experimental approaches in order to understand and improve animal welfare in the “real world” [14–16]. This shift has practical implications as it is difficult to impose strict experimental conditions in working contexts such as farms or zoos without interfering with daily operations [17]. It therefore also places some limitations on the assessment methodologies that can be used. However, conducting research in non-experimental settings provides the important benefit of offering a methodological solution to the fact that the causes of welfare problems are often multi-factorial. For example, skeletal disorders (e.g. lameness, vertebral deformity) are significant welfare problems in commercial broiler (meat) chicken production. The incidence and severity of these problems can be affected by genetics, nutrition, lighting, stocking density, litter management, and the presence of various infectious organisms in the hatchery, breeding or rearing facility [18]. It is virtually impossible to study all of these factors and their interactions using traditional experimental approaches.

Unlike traditional experimental approaches that try to understand cause-and-effect by limiting variability due to non-experimental factors, epidemiology takes advantage of the fact that there is variation across populations (e.g. houses on a farm, farms, transport trucks, slaughter plants) and uses that variation to examine patterns. Steps required to conduct an epidemiological analysis of animal welfare include identifying the welfare problems of interest (outcomes) and the housing and/or management factors that could affect the incidence or severity of those problems (inputs, or risks). The inputs and outcomes are then measured at multiple sites, and multivariate models created to determine the relationships among them. Epidemiological models are correlational and do not definitively establish the cause(s) of specific welfare problems. They do, however, provide information about the overall prevalence of welfare problems and the relative importance of each of the various risk factors that are found to affect that prevalence. These data can then inform housing and management changes to reduce the risk of those problems occurring.

Despite being a relatively new field, there is now a significant research base for animal welfare science [6]. It can thus be considered to be a well-developed field of inquiry, which has led to many advances not only in our understanding of the basic biology of animals as it relates to their welfare but in methods to improve animal welfare via changes in housing, handling, breeding, and management. While the majority of existing research is focused on farm animals (see for example [5, 19]), the methods and principles of the field have more recently been extended to other settings, including zoos.

## Animal Welfare Science in Zoos

Animal welfare science as applied in zoos aims to determine the best possible husbandry practices to promote the welfare of individuals of all species. Because of the wide diversity of animals housed in zoos this has yet to be accomplished for the vast majority of species, and by and large caring for zoo animals is still more of an art rather than evidence-based [20]. While there have been intra-and inter-individual comparative studies of welfare outcomes such as stress responses, reproduction, husbandry, health and abnormal behaviors with zoo species, they are heavily weighted towards mammals [21].

Zoos present challenging research settings because species are housed in small numbers under conditions that vary in different institutions. In addition, it is often difficult or impossible to conduct experimental manipulations of environmental conditions in zoos that are thought to affect welfare, such as space or specific features of exhibit construction. The exception is research focused on ‘environmental enrichment’, which involves making changes to an animal’s environment that provide the animal with added stimulation, choice or control [22]. Environmental enrichment research has proliferated in zoos over the past three decades because the experimental manipulations are non-invasive and cost effective, and the effects are also evaluated largely non-invasively by observing behavioral changes such as reduced stereotypy, increased activity, increased behavioral diversity and positive social behaviors, or by assessing stress responses via fecal glucocorticoid measurement. Most enrichment research in zoos focuses on the proximate goals of giving animals more choices, more complex environments, more naturalistic surroundings with natural behavioral opportunities, and less stressful living situations [22]. For example, Wielebnowski et al. [23] demonstrated that adding climbing structures and increasing hiding places in the enclosures of clouded leopards (*Neofelis nebulosa*) reduced fecal corticoid levels in individuals of this arboreal species, indicating that this specific type of environmental change had positive benefits to welfare.

Zoo animal welfare science is evolving into its own specialty with unique goals since individual animals in modern zoos are expected not only to thrive, but also (unlike farm animals) to lead long, high-quality lives while serving as conservation ambassadors for their wild counterparts. Currently, the strongest contribution by zoos to animal welfare science is on topics like species differences in predisposition to specific welfare problems and limitations of adaptability to captivity (e.g. [24, 25]), development of tools for individual welfare assessment (e.g. [26]), and the impact of keeper-animal interactions [27–30] and visitors on animal welfare [31, 32]. Additionally, zoo animal welfare scientists collaborate with academic researchers to study differences in animal personalities and their implications for biological functioning in zoo environments [33–36]. In addition, because of the small numbers of individuals housed in zoos and the variation between zoos in housing and management, there has been increasing interest in improving the predictive power of zoo animal welfare research by conducting multi-institutional, multi-disciplinary studies [37–38]. Such studies have elucidated the relationships between enclosure size, nest box provision and cub-rearing success in red pandas [39], enclosure size and breeding success in black rhinoceros [40], and enrichment, group size, cage size, enclosure design and stereotypic behavior in bears [41, 42].

## The Need for Zoo Elephant Welfare Research

Although elephant welfare and conservation are clear priorities for zoos, several authors [43–45] have identified key gaps in knowledge of elephant biology and care. These authors have called for large-scale empirical research projects on zoo elephants that provide unambiguous, welfare-relevant data. Evidence that welfare is not optimal among zoo elephants includes reported high rates of stereotypic behavior [46], a high prevalence of ovarian acyclicity [47, 48], various health issues such as obesity, tuberculosis, herpes, and foot problems [49–52], and compromised survivorship [53]. However, there was a lack of knowledge about the factors that predict these indicators of poor welfare. With funding from the (U.S.) Institute of Museum and Library Services, our research team undertook the largest zoo animal welfare research project ever conducted with extant animals North American zoos: a multi-institutional, multi-disciplinary, epidemiological assessment of a range of welfare indicators for 255 elephants in relation to husbandry and housing at 68 zoos accredited by the Association of Zoos and Aquariums (AZA).

The project was initiated by convening a diverse team of experts including zoo and university affiliated scientists specializing in behavior, endocrinology, and physiology, elephant care professionals, and veterinarians. This panel developed a framework that defined a range of welfare outcomes by adapting criteria established by the European Union Welfare Quality (WQ) project for farm animals ([54], see [16] for more details on project development) and identified a range of welfare inputs and outcomes, which were subsequently refined based on validity and feasibility of data collection. This framework supported a targeted, yet multi-factorial, approach, as illustrated by the studies reported in the papers in the collection in this volume.

## The Collection: Epidemiological Investigations of North American Zoo Elephant Welfare

**Fig 1:**
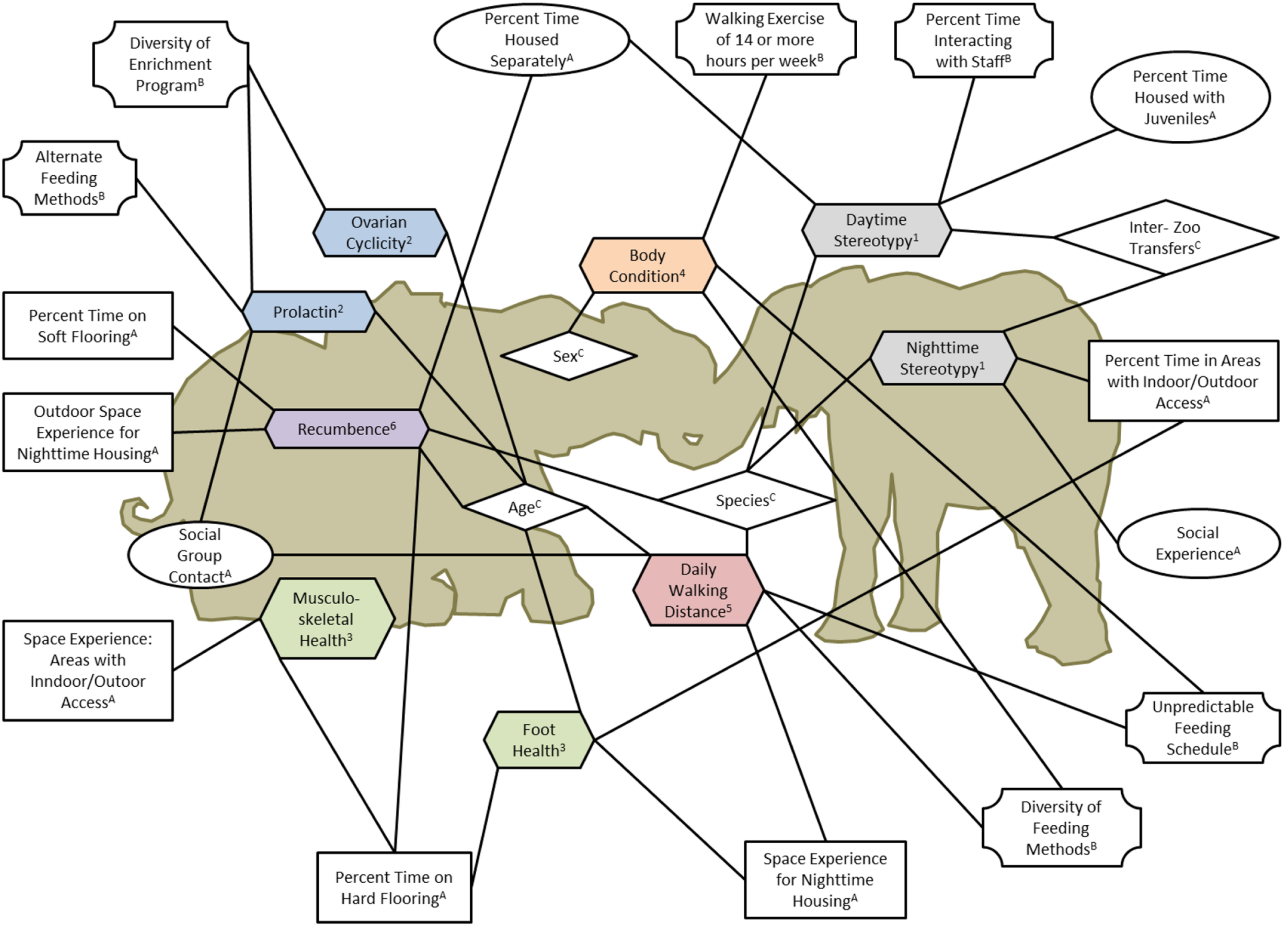
The associations between each welfare outcome and its independent variables are represented by connecting lines. Independent variables are grouped categorically by icon shape as follows: Housing (Rectangle); Social (Oval); Management (Octagon); Life History/Demographic (Diamond) factors. For more information on the welfare outcomes studied, please see: (1) Greco et al. [60]; (2) Brown et al. [63]; (3) Miller et al. [58]; (4) Morfeld et al. [59]; Holdgate et al. [61]; Holdgate et al. [62]. For more information about the independent variables tested, please see: (A) Meehan et al. [55]; (B) Greco et al. [56]; and (C) Prado-Oviedo et al. [57]. Note that this diagram displays non-directional associations. For details on the directionality and magnitude of associations, see Fig 2.

We have organized this collection of nine papers to reflect the methods of epidemiological studies. The first three papers provide an introduction to the independent variables and characterize the aspects of animal management that were hypothesized to be influential to welfare, including measures of housing and social management [55], life history and demographics [56] and feeding, training, enrichment and exercise [57]. The remaining six papers focus on eight physical [58, 59], behavioral [60–62], and physiological [63] outcomes for which we assessed a defined population of zoo elephants. These papers provide information on the distribution of welfare outcomes across the population, and present multi-variable models designed to determine associations with independent variables. Over 50 independent variables were tested across all six papers. Of these, 18 unique variables were found to have significant associations with one or more welfare indicators. Fig 1 presents a graphic representation of the connections between each welfare outcome and its associated independent variables. In the sections that follow, we discuss the themes that emerged when we considered these connections collectively across the studies.

## Sociality

Both African and Asian elephants exhibit complex and elaborate patterns of sociality in the wild. Thus, it is unsurprising that social variables were important predictors of three of four behavioral indicators of elephant welfare—daytime stereotypic behavior, nighttime stereotypic behavior and walking distances—and were a risk factor for hyperprolactinemia, an endocrine system dysfunction. For example, Greco et al. [60] characterize five of the seven unique variables in their models that predicted stereotypic behavior rates as social variables. These include the amount of time the elephants were housed alone (which increased risk) as well as the those that decreased risk such as the percent of time spent with juveniles, percent of time spent with human caretakers and overall social experience—a measure of that weighs the size of social groupings by the time spent in each group [55]. The authors conclude that the connection between stereotypy performance and sociality, both with conspecifics and with humans, is “*particularly compelling given the highly social nature of elephants and their propensity to form strong social bonds”* [60], and recommend that zoos consider social factors in their efforts to reduce the development and performance of these behaviors.

Meehan et al. [55] present a novel metric for assessing zoo elephant social management titled Social Group Contact. This variable reflects the facts that, in most zoos, herds are divided into various sub-groups, and that individual elephants generally spend time in more than one of these sub-groups. As the number of social groups increased for African female elephants so did their risk of being hyperprolactinemic. Brown et al. propose that this link could be related to the role prolactin plays in stress responses, particularly socially related stressors, and suggest that “*for female African elephants*, *not being in a stable social group may be a stressor that elicits an increased prolactin response*” and that “*social management practices that include dividing the herd into multiple social groups and housing elephants in a variety of social configurations may yield a more demanding social environment to which they respond with elevated prolactin*” [63].

Studies of other social species have shown that isolation, exposure to groups of unnatural size or composition, or repeated disruption of established social groups have detrimental effects on welfare [64,65] and conversely, that an appropriate social milieu can promote positive welfare [66,67]. The papers in this collection indicate that, for zoo elephants, good welfare is supported by spending more time in larger, stable social groupings that include both juvenile and adult elephants, and reducing time spent in isolation.

## Housing

The topic of space is rarely absent from discussions about zoo elephant welfare. Space is generally a limited resource for zoos and expansion of exhibits can be a lengthy and costly process. It is important, therefore, to better understand the influence exhibit size has on the welfare of zoo elephants so that decisions about how best to manage spatial resources can be properly informed. Measuring space in zoo elephant environments is complicated by the fact that zoo exhibits rarely function as contiguous units, but rather are subdivided with the individual spaces used separately or in combination to house groups of elephants for varying amounts of time throughout the day and night. In Meehan et al. [55], the authors propose a method for measuring individual elephants’ experience of space using a variable that weighs the area of individual environments by the time spent in each environment. The resulting variable, Space Experience, captures the concurrent effects of area and time in a measure of space. Space Experience, and its derivations, were tested in all outcome models with few resulting associations. Increased Space Experience in outdoor areas during the night at was associated with African elephants spending more in recumbent rest [62], but measures of space were not identified as risk factors for stereotypic behavior [60], obesity [59], or female reproductive dysfunction [63]. Contrary to our hypotheses, increased space was negatively associated with walking distances [61] and positively associated with the incidence of foot abnormalities [58], although in both cases the effect size was small.

The configuration of the space provided was found to be important to both the performance of stereotypic behavior [60] and foot health [58], but with opposite directional effects. Specifically, spending time in housing areas that allowed elephants to move freely between indoor and outdoor spaces reduced the risk of stereotypic behavior performance, but increased the likelihood of foot problems. Providing indoor/outdoor housing may support an elephant’s ability to make choices about their environment, which supports behavioral health [56], but may also expose elephants to environmental features (thresholds, gates) or temperature fluctuations that could contribute to foot problems [58]. The fact that a single variable can have differential effects demonstrates the importance of assessing multiple welfare indicators simultaneously.

Flooring type was hypothesized to be important to multiple welfare outcomes and Meehan et al. [55] present data on the time spent by individual elephants in environments that had 100% coverage of either hard (concrete or stone) or soft (sand, rubber padding, grass) flooring. Time spent in environments with 100% coverage of hard flooring was the greatest risk factor for musculoskeletal health problems and the second greatest risk (after advancing age) for foot health problems. Miller et al. [58] propose that elephants’ unique pedal and limb anatomy and large body mass may predispose them to foot and joint conditions and recommend limiting exposure to hard flooring as a primary strategy for reducing the incidence of foot and musculoskeletal problems in the studied population.

In addition, Holdgate et al. [62] demonstrate strong associations between flooring and patterns of elephant recumbence (lying rest). Time spent on hard flooring was associated with decreased resting time for African elephants and time spent on soft substrates was associated with increased resting time for Asians. The authors point to similar findings from studies of lying rest in cattle and suggest that hard flooring is not conducive to recumbence and *“may be contributing to sleep deprivation or sleep disturbance*, *or causing stress or frustration in animals that are reluctant to exhibit natural resting postures*.*”* [62].

Taken together, the results of our analyses indicate that the size of an exhibit plays a secondary role in supporting optimal welfare of zoo elephants, at least based on our outcome measures. It is, however, important to consider space configuration as well as flooring. In addition, adequate space is necessary to support larger social groupings as well as robust feeding and enrichment programs (see discussion that follows). While this investigation of space as a predictor of zoo elephant welfare is the most thorough to date, it was limited to the range of exhibit sizes at participating North American zoos, and future studies incorporating larger areas could potentially find associations between space and welfare outcomes.

## Management Factors

Although feeding, training, exercise, husbandry and environmental enrichment programs are all required elements for zoos accredited by the Association of Zoos and Aquariums (AZA), there has been little previous research that measures these practices or tests their associations with elephant welfare. These practices may be the most readily modifiable aspects of elephant care (as compared to exhibit remodel or social group changes, for example), and so research demonstrating their influence on welfare outcomes provides a significant opportunity to improve management, and hence elephant welfare. Greco et al. [56] provide an overview of elephant management and present over 20 different variables that they used to quantify the range of practices in zoos. The significance of these management variables to welfare outcomes is addressed in several papers.

For example, Brown et al. [63] describe two reproductive health issues that are particularly relevant to the population of African female elephants: ovarian acyclicity and hyperprolactinemia. Both of these conditions contribute to reduced fertility. Interestingly, the factors that were the most important in reducing the risk of reproductive problems were feeding and enrichment programs. For example, Enrichment Diversity, an index that quantified the variety and frequency of environmental enrichment provision, had a strong protective effect for both cyclicity status and prolactin level, and the use of alternate feeding methods (when food was presented by hanging it above the enclosure, hiding it within the enclosure or putting it in an enrichment device such as a puzzle feeder) had an additional protective effect against hyperprolactinemia. The authors interpret these findings in the context of reproduction in wild African elephants. They explain that wild African elephants have a flexible reproductive strategy that relies on assessment of the quality of a range of environmental cues including, but not limited to, food and water availability to optimize the timing of reproduction, and suggest “*for African elephants in zoos*, *it may be that low diversity of enrichment*, *including feeding options*, *signals an environment poor in resources*, *even though food and water are not limited”* [63]. It follows, then, that zoos with robust enrichment and feeding programs may provide environments that are perceived as rich in resources and to which elephants respond with optimal pituitary and/or ovarian function.

Body condition is another welfare outcome for which management practices were primary risk factors. Poor body condition is an important health concern for zoo elephants because of plausible associations with conditions such as such as cardiovascular disease, arthritis and foot problems, and ovarian cycle abnormalities [68,69]. Morfeld et al. [59] report that the majority of the 240 elephants they assessed were overweight or obese. The authors tested a variety of non-nutritional aspects of feeding programs such as frequency, method and timing of presentation as well as a range of variables describing exercise programs as risk factors for elevated body condition. Two of these had important protective effects—unpredictable feeding schedules and staff-directed walking exercise. Given that only 20% of the zoos studied utilized unpredictable feeding schedules [56], and the majority of elephants participated in less than one hour of walking based exercise per week [56], there is significant potential for addressing poor body condition in this population by applying the management changes indicated by these analyses.

## Demographics and Life History

In addition to associations with housing and management factors, an individual’s welfare can be linked to demographic factors such as age, sex, and species or life history events such as transfers between zoos, and Prado-Oviedo et al. [57] provide a thorough overview of a variety of these types of variables. Species, sex, age and origin (imported or zoo-born) were tested as independent variables and confounders in all outcome analyses, and while origin was not predictive of any welfare outcomes, species, sex and age were significant factors in several models. Female African elephants were more likely to be affected by ovarian acyclicity and hyperprolactinemia than Asian females, and rates of stereotypic behavior [60], patterns and durations of recumbent rest [62], and walking distances [61] differed between the species. With respect to sex, female elephants were more likely to be overweight or obese than their male counterparts [59]. Age was also a risk factor for reproductive dysfunction in females [63] and joint abnormalities [58]. Unlike management factors, age, species and sex are not directly modifiable, but knowledge of how individuals with different demographic profiles may be at greater risk for specific welfare issues can inform management strategies so as to potentially mitigate these predispositions. For example, knowing that Asian elephants are much more likely than Africans to develop stereotypic behaviors may lead zoos that house this species to prioritize early interventions by limiting time spent housed alone and increasing overall social experience.

Prado-Oviedo et al. [57] also characterized the life histories of elephants with respect to social events such as inter-zoo transfers. These events are important in the context of elephant welfare because both Asian and African elephants have complex social systems centered around matrilineal core groups composed of genetically related adult females and their dependent offspring [70, 71]. The authors report that a majority of the elephants had experienced at least one inter-zoo transfer in their lifetime, with a range within the population of 0–10 transfer events. Greco et al. [60] found that the number of transfers experienced was an important risk factor for the performance of stereotypic behavior and suggest that factors such as travel, unfamiliar surroundings, and social separation may be the links between transfers and stereotypic behavior, although more research is needed to explicitly test for causality.

**Fig 2:**
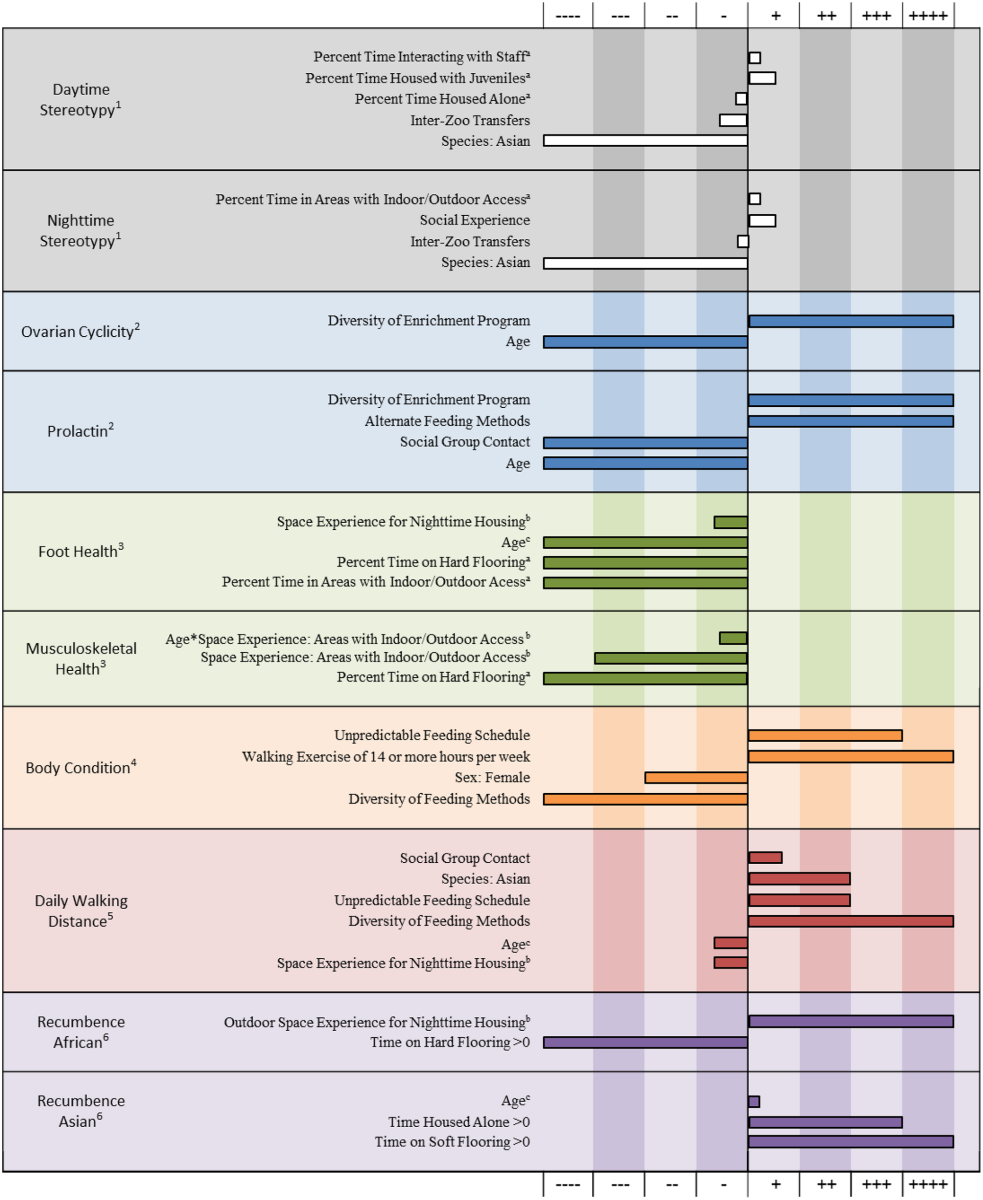
Relative impact of input variables for each model described in the papers that comprise this collection. Beta values were standardized by [Beta Value] / [Maximum Beta Value] for each individual model. As noted, some Beta values have been scaled as follows: (a) per 10% time; (b) per 500 square feet of space; (c) per 5 years of age. Directional effects were normalized and are represented as gradations of positive and negative associations with welfare based on association with welfare outcomes. Shown with a positive direction are factors that decreased the risk of performing stereotypic behavior (1), [60]; decreased the odds of ovarian acyclicity (2), [63]; decreased the odds of being hyperprolactinemic (2), [63]; decreased the risk of persistent foot problems (3), [58]; decreased the odds of musculoskeletal problems of the limbs (3), [58]; decreased the odds of high body condition scores (i.e. overweight or obese) (4), [59]; were associated with increased daily walking distance (5), [61]; and were associated with increased recumbence (6), [62]. Comparisons of magnitude of effect should be made within outcome only.

Epidemiological analyses allow for the detection of relationships between animal welfare outcomes and “real world” environmental variation. This is accomplished by building multi-variable analyses that model the combined effects of the factors with significant associations. Fig 2 provides a comprehensive summary of the models included in this collection and illustrates the direction and magnitude of effect for each factor highlighted in Fig 1. While looking across models (Fig 1) reveals themes with respect to how independent variables influence welfare, looking within models provides insight into to the role each variable plays with respect to each outcome, which can inform strategies to apply this information to the practice of elephant care.

## Future Directions

The papers presented in this collection contribute significantly to the field of zoo animal welfare science with respect to both its process and its products. Collectively, the studies established a model for multi-institutional zoo animal welfare research by leveraging data contributed by many individual elephants from a diverse array of zoos. This process yielded specific metrics and identified risk factors that can inform progress toward population-level and individual elephant welfare goals. Although epidemiological analyses of the kind presented in this collection are by nature only correlational, the results of these studies provide a strong starting point for additional experimental work as they point clearly toward several aspects of housing and management that were found to be strongly associated with the welfare of zoo elephants. Importantly, many of the variables highlighted by this research are modifiable, and can be monitored to determine if the predicted effects on welfare outcomes are subsequently achieved. Forthcoming from the data collected in this study will be additional analyses of health and disease, keeper-elephant relationships, and individual differences in elephant personality in relation to welfare outcomes. Looking ahead, we envision future studies that build upon this model by refining the metrics we have presented, incorporating additional and emerging measures of animal welfare, and integrating experimental components to further elucidate the complex and important connections between the daily lives of zoo animals and their physical and psychological states.
